# Metropolitan Hospital Sunday Fund

**Published:** 1901-02-16

**Authors:** 


### The Institutional Workshop.

### METROPOLITAN HOSPITAL SUNDAY FUND.

A COUNCIL meeting of the Metropolitan Hospital Sunday Fund was held at the Mansion House on Thursday, 7th inst., the Lord Mayor presiding. The business before the Council was: To consider a resolution of sympathy and condolence consequent on the death of the Queen; and to fill a vacancy on the Committee of Distribution. A letter had been received from Mrs. Creighton and her family, thanking the Council for their kind expression of sympathy with them on the death of the Bishop of London, and a similar one from Mrs. Sedgwick Saunders, on the death of Dr. Sedgwick Saunders.

The LORD MAYOR addressed the Council on the death of the Queen, alluding especially to the interest always taken by her Majesty in alleviating suffering and distress, an interest shown more than in any other way in connection with hospitals.

The Ven. Archdeacon SINCLAIR, in proposing the resolution of condolence, said Queen Victoria had devoted her life to benefiting her fellow-creatures; there had been opportunities in her long reign for the exhibition of sympathy with suffering, and the example exercised in such a direction had been a most momentous power for good. The tide of sympathy and of the desire to alleviate suffering, which had set in during the last half-century, was a most delightful sign, and he thought it not too much to say that this impulse had been originated by her late Majesty herself. During her pleasant sojourns in her Highland home she had delighted to visit the aged and read to them; and quite recently she had visited the Military and Naval Hospitals to cheer those who had lost limbs in her service and the service of her country; and her sympathy with the sorrows of those who had lost relatives in the field was well known. It was not needful, when such a chorus of sorrow and regret was heard from pulpit and town-hall, to enter deeply into the reasons for grief which all felt. But this Council desired to record its gratitude for her late Majesty's Christian-principled conduct throughout her wide dominions; gratitude shared by people of other creeds—the great Mahomedan population of India, for example, felt her influence to be one of greatest blessing: they had never seen the Queen, but they loved her and cherished her memory. The Archdeacon then alluded to the wonderfully active share taken by his Majesty the King in the promotion of the same objects as those which the Queen had had at heart; and he congratulated the parallel fund to their own—the Prince of Wales's Fund—on the legacy it had just received, and hoped that the efforts being put forth would increase in extent and usefulness under his Majesty's encouraging guidance.

The resolution was seconded by Dr. ADLER, who said he was sure it was the wish of the Council to accentuate the fact that all united, without distinction of race or creed, in sorrow for the nation's loss. "We of our religion," said the speaker, "have had the privilege from our pulpits of bearing our record." Nothing had so pre-eminently distinguished her reign as the bringing together of what Lord Beaconsfield had called "two nations"—the rich and the poor. When the Queen was bowed down by infirmities, she had herself wheeled among her soldiers, so that they forgot their wounds in her queenly smile of gratitude. They had had opportunities of noting the extraordinary kindliness of heart, and wisdom in guiding, of her successor; the desire of the parallel fund to their own was not to rival it in the slightest degree, but to improve and benefit the institutions which it was the aim of both to help.

The Rev. W. HARDY HARWOOD supported the resolution on behalf of the Nonconformist churches, whose aim had always been to teach that religion and loyalty were wedded together; no section of the community felt the loss more deeply; they felt that they had lost a great ally.

Sir HENRY BURDETT said he thought that the noblest and truest epitaph to her Majesty was spoken by the cabman who drove him home on the night the Queen died: "Ah, Sir, her life was an example to us all." It was a lesson which he hoped the clergy, with their special opportunities, would drive home. As a council dealing with hospitals, it was well to remember that out of 4,000 proposals to commemorate the Diamond Jubilee, the Queen let it be known that it was her wish to be associated with the Prince of Wales's Fund.

In putting the resolution, the LORD MAYOR said the statement that the Queen's death was the "common loss of all humanity," was proved by the fact that among many telegrams that had come to the Mansion House, one of the first was from Buenos Ayres; only the day before this meeting one was received from Cephalonia, and that day one had come from Messina, in Sicily, expressing the warm feelings of the Italians.

The resolution, with one or two amendments, was adopted as drafted by the Ven. Archdeacon Sinclair, to be engrossed and presented to his Majesty on behalf of the Council.

The appointment of Sir William S. Church, M.D., was then made, to fill the vacancy caused by the death of Dr. Sedgwick Saunders; this was proposed by Sir JOHN SAVORY and seconded by Lord STAMFORD. Sir WILLIAM CHURCH accepted the appointment, and placed his services, and long experience of the Metropolitan hospitals, at the disposal of the Council.

Owing to the serious indisposition of the secretary, Mr. H. N. Custance, who had been ordered a month's sea-voyage by his physician, an informal vote was passed according Mr. Custance the required holiday.

"TO THE KING'S MOST EXCELLENT MAJESTY.

"The loyal and dutiful address of the Presidents, Vice-Presidents, Treasurers, and Council of the Metropolitan Hospital Sunday Fund:

"MOST GRACIOUS SOVEREIGN:

"We, the Presidents, Vice-Presidents, Treasurers, and Councillors of the Metropolitan Hospital Sunday Fund humbly approach your Majesty to offer our heartfelt condolence on the demise of her Majesty your noble and illustrious Mother, our late gracious and beloved Sovereign, whom we mourn with profound sorrow. We are specially entitled to express that deep grief which we share in common with our fellow-subjects throughout the Empire, by reason of the lifelong sympathy of her late Majesty with those objects which it is our aim to promote, and the great interest which she evinced for the cause of Hospitals during the whole period of her long and glorious reign. It is a consolation to us, as it is to all our fellow-subjects, to approach your Majesty on your accession to the throne of your ancestors. We most humbly tender to your Majesty our condolence and respectful homage and heartiest wishes for your reign. It is a source of high gratification to us that your Majesty in 1897 founded a parallel movement known by your own title when heir to the throne, in commemoration of the Diamond Jubilee of your illustrious Mother's great reign, and that your Majesty throughout your life has evinced the warmest interest in all that promotes the moral and material welfare of your subjects. All concerned with this Fund will desire to unite with us in assuring your Majesty of our sincerest prayers that it may please God long to preserve your Majesty to rule over a loyal, united, and religious people, and that all prosperity and well-being may attend your Majesty's reign."

